# Assessment of Rapid Antigen Diagnostic Tests at Mass Events: Identifying Optimal Floor Plan Configurations for Enhanced Efficiency

**DOI:** 10.3390/healthcare12232375

**Published:** 2024-11-26

**Authors:** Anas A. Khan, Ahmad F. Turki

**Affiliations:** 1 Emergency Medicine Department, College of Medicine, King Saud University, Riyadh 11362, Saudi Arabia; anaskhan@ksu.edu.sa; 2 Global Center of Mass Gatherings Medicine, Ministry of Health, Riyadh 11451, Saudi Arabia; 3 Electrical and Computer Engineering Department, Faculty of Engineering, King Abdulaziz University, Jeddah 21589, Saudi Arabia; 4 Center of Excellence in Intelligent Engineering Systems (CEIES), King Abdulaziz University, Jeddah 21589, Saudi Arabia

**Keywords:** COVID-19 testing, queueing theory, *MAP/PH/c* model, mass gatherings, operational efficiency, agile management, system throughput, public health emergency management, antigen testing, process optimization

## Abstract

Background/Objectives: The COVID-19 pandemic underscored the urgent need for rapid, efficient testing methods at large-scale events to control virus spread. This study leverages queueing theory to explore how different floor plan configurations affect the efficiency of Rapid Antigen Diagnostic Test (RADT) centers at mass gatherings, aiming to enhance throughput and minimize wait times. Methods: Employing the *MAP/PH/c* model (Markovian Arrival Process/phase-type service distribution with c servers), this study compared the operational efficiency of RADT centers using U-shaped and straight-line floor plans. The research involved 500 healthy participants, who underwent the RADT process, including queue number issuance, registration, sample collection, sample mixing, and results dissemination. Agile management techniques were implemented to optimize operations. Results: The findings demonstrated that the U-shaped layout was more efficient than the straight-line configuration, reducing the average time from sample collection to results acquisition—1.6 minutes in the U-shaped layout versus 1.8 minutes in the straight-line layout. The efficiency of the U-shaped layout was particularly notable at the results stage, with statistically significant differences (*p* < 0.05) in reducing congestion and improving resource allocation. Conclusions: The study confirms the feasibility of implementing RADT procedures at mass gatherings and identifies the U-shaped floor plan as the optimal configuration. This layout significantly enhances testing efficiency and effectiveness, suggesting its suitability for future large-scale testing scenarios. The research contributes to optimizing mass testing strategies, vital for public health emergency management during pandemics.

## 1. Introduction

Diagnostics and screening were vital in responding to the COVID-19 pandemic; rapid and efficient testing methodologies were necessary for quick screening, particularly at large-scale events [1]. Rapid Antigen Diagnostic Tests (RADTs) for COVID-19 are designed to detect the presence of viral proteins (antigens) expressed by the SARS-CoV-2 virus in samples typically taken from a person’s nasal cavity using swabs [2]. Manufacturers of RADTs indicate that these tests can take up to 15 min to deliver the test results, making them particularly valuable in environments that demand rapid decision-making, such as airports and mass gatherings [3]. RADTs are designed to detect specific antigens associated with the SARS-CoV-2 virus through a methodical process that begins with sample collection and ends with display of the results [4]. The procedure initiates with the collection of a nasopharyngeal swab, which involves obtaining respiratory epithelial cells from the posterior nasopharynx, where the viral load is typically concentrated in infected individuals [5,6].

Following collection, the swab is immersed in a specialized reagent solution that serves multiple functions: it lyses viral particles to release their components, stabilizes viral proteins, and enhances the detectability of viral antigens [7].

The prepared sample is then applied to a test strip embedded with a nitrocellulose membrane pre-coated with antibodies that are specific to SARS-CoV-2 antigens [6]. As the sample migrates along the strip via capillary action, any viral antigens present will bind to these immobilized antibodies [7].

The accuracy of RADTs for SARS-CoV-2 is quantitatively evaluated through two principal metrics: sensitivity and specificity [8]. Sensitivity, defined as a test’s ability to identify infected individuals correctly (its true positive rate), exhibits considerable variability among RADTs, with these values ranging from approximately 50% to above 90% [8]. This range depends on several critical factors: the temporal proximity to symptom onset, which correlates with peak viral shedding; the viral load present in the collected specimen; and the precision of the sample collection technique employed [8].

Specificity, which measures a test’s ability to identify uninfected individuals correctly (its true negative rate), typically maintains a higher consistency across various RADT platforms, often exceeding 95% [8]. This high level of specificity indicates a low incidence of false positives, where a test erroneously suggests the presence of SARS-CoV-2 [9]. The robust specificity of RADTs underscores their utility in minimizing the likelihood of unnecessary quarantine or additional diagnostic procedures for individuals wrongly identified as infected [9].

A study by the University of Illinois at Urbana-Champaign (UIUC) encapsulates the importance of efficient testing infrastructure [10]. In the UIUC’s study, a discrete event simulation model was utilized to optimize the design of saliva-based COVID-19 testing stations [10]. This model was crucial in determining the optimal number of machines and operators required, as well as their efficient allocation at various workstations, based on daily testing volumes and resource availability [10].

A study conducted by Conor G. McAloon and colleagues has been instrumental in shaping the strategic deployment of RADTs at large-scale public events [2]. This research provides crucial insights into how RADTs can effectively be used to screen attendees for SARS-CoV-2 infection, offering a practical solution for enhancing public health safety during mass gatherings [2]. Conor G. McAloon et al.’s study assessed the potential prevalence of infectious individuals under varying epidemiological scenarios by employing a simulation approach [2]. Their findings reveal the conditions under which RADTs yield the most value, demonstrating their ability to significantly reduce the risk of viral transmission among large groups [2].

Our study is driven by employing the principles of a queueing-theory-based approach. [11]. By applying an analytical approach to queue dynamics, this study aims to substantially reduce waiting times, thereby accelerating the testing process and bolstering public health and safety during mass gatherings. Queueing theory investigates the interactions between customers and those providing services (servers) [12]. The daily experience of waiting in line plays a critical role in managing the flow of customers, especially when resources are limited [12]. Queuing theory offers actionable insights by focusing on essential variables: the average number of customers in the system (*L*), the arrival rate (*λ*), and the time spent in the system (*W*) [13]. Central to this theory is Little’s Law, which facilitates the estimation of crucial metrics like waiting times and queue lengths from minimal input data [14].

Within the context of RADTs, the *MAP/PH/c* queueing model—which generalizes Poisson’s process for arrival rates and allows for flexible phase-type distributions for service times—offers a more realistic framework for analyzing the system dynamics [13]. In this model, “*c*” represents the number of service channels or testing stations, and it intricately accounts for the complexities of multiple servers and setup times. This approach enables detailed calculations of the arrival rates, the service rates, and the number of lanes required to optimize efficiency and ensure smooth operations, even under varying conditions [15].

We designed proposed U-shaped and straight-line floor plans to streamline operations, supported by systematic data collection and analytical assessment of the different testing scenarios. Furthermore, advanced analytics and computational models have shown significant potential in predicting outcomes across various medical fields [16].

## 2. The Method

This research was designed to assess the processing speed of RADTs in a large-scale setting. We chose the widely available Abbott Panbio COVID-19 RADTs (Abbott Laboratories, Chicago, IL, USA) [17] for this analysis. The aim was to determine how different floor plan arrangements could enhance the flow and expedite the processing of the test kits among a diverse group of event attendees.

This study involved 500 healthy participants aged between 17 and 55, including 250 females, with a mean age of 32 ± 8.4 years. Five members of staff with medical backgrounds worked at each stage. All individuals gave their informed consent before participating in this study. The choice of the sample size for this study was guided by the need to ensure robust statistical power and representation while balancing the practicalities of managing a large-scale testing event.

This study employed the *MAP/PH/c* model to accurately capture the variability in the arrival patterns and service times across multiple stages. Unlike simpler queuing models, the *MAP/PH/c* model accommodates non-exponential service times and fluctuating arrivals, providing a more realistic representation of the testing process. This approach allowed us to evaluate the system’s stability, throughput, and performance across two distinct floor plan layouts: U-shaped and straight-line.

**Figure 1:**
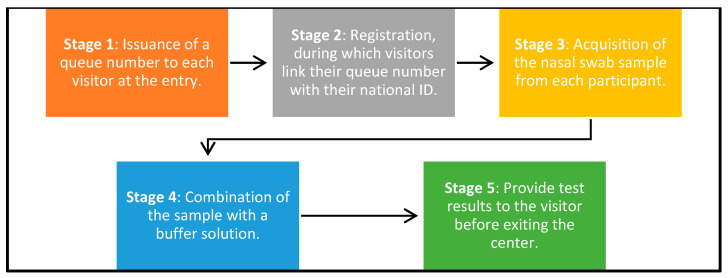
Five test stages.

This study was conducted in a controlled test center environment organized into two-floor plans. The first configuration adopted a U-shaped layout, whereas the second was designed in a straight-line format. Each configuration was segmented into five crucial stages, as illustrated in Figure 1:

1. Stage 1—Registration: Participants receive a number sticker upon arrival.
2. Stage 2—Sample collection: Swabs are collected from the participants.
3. Stage 3—Sample mixing: Samples are immediately processed to reduce delays.
4. Stage 4—Result processing: The test results are prepared.
5. Stage 5—Result dissemination: Participants receive their results and exit the facility or receive further instructions.

**Figure 2:**
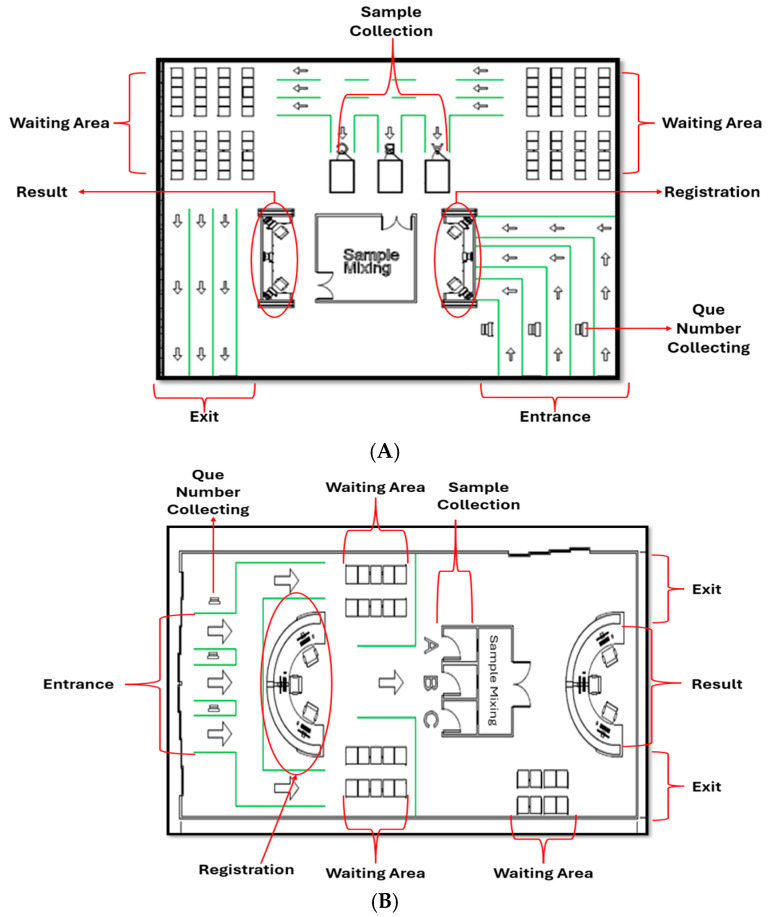
(**A**) Floor plan 1 (U-shaped) and (**B**) floor plan 2 (straight line). Both floor plans include three testing lanes labeled A, B, and C at the sample collection station. All lanes function identically, ensuring consistent testing procedures across different configurations.

The floor plans, depicted in Figure 2, included three channels with strategic placements of entrance kiosks, waiting areas, sample mixing zones, result kiosks, and exits. Although waiting areas were provided, they were not utilized during the experiment due to the smooth flow of the participants throughout the process.

All of the simulations and floor designs were designed using Autodesk Revit software (Autodesk Revit, Autodesk, Newton, MA, USA) [18].

Upon entering the testing center, each subject was given a number sticker, promoting sequential progression through the designated testing stages and enhancing the procedural methods. The data collection was carefully managed using a shared Google Sheet (Google LLC, Menlo Park, CA, USA) [19], where the study’s data collectors recorded the timestamps for each stage for every participant, ensuring synchronized timekeeping and precise data collection. The participants were guided to specific seats for sample collection, followed by immediate processing of the samples in the adjacent mixing area to reduce wait times and streamline the process; after processing, the results were relayed to the participants at a designated result kiosk; and depending on these outcomes, the participants were either directed to leave the center or provided with additional instructions.

The data acquisition was thorough, capturing detailed records on the arrival rates, service times, and setup duration for each layout. Appropriate queuing models, specifically *MAP/PH/c*, were implemented to assess the system’s efficiency under various conditions, considering the setup times. We used R software (R Software Technologies LLC, San Francisco, CA, USA) [20] to calculate the D_0_ and D_1_ matrices for each stage:

- D_0_ matrix: represents internal service transitions and completion rates at each stage.
- D_1_ matrix: describes arrival transitions, determining the arrival rate (*λ*).

R was chosen due to its efficient handling of complex matrix operations.

These matrices were then used as inputs in MATLAB R2022b (MathWorks, Natick, MA, USA) [21] to implement the *MAP/PH/c* model, enabling us to achieve the following:

- Calculate traffic intensity (ρ) to maintain system stability (ρ < 1);
- Model the throughput, queue lengths, waiting times, and blocking probabilities under various conditions.

Agile management techniques were employed, particularly in the distribution of the tasks among the testing team. Each team member was responsible for a specific portion of the testing process, allowing for concentrated expertise and rapid execution of tasks.

## 3. Results

A statistical analysis using a *t*-test revealed no statistically significant differences in the time spent at stages 1 through 4 between the two floor plans (*p*-value > 0.05). However, a significant difference was observed at stage 5 (*p*-value < 0.05), suggesting that the layout configuration has a notable impact on the efficiency of dissemination of the results. The significant difference at stage 5 can be attributed to the varying levels of congestion and resource allocation in the result dissemination stage between the two layouts. The U-shaped layout likely provides a more streamlined flow and better management of the participants during this stage, reducing the wait times and service delays. Conversely, the straight-line layout may lead to more bottlenecks and inefficient handling of results, contributing to a longer time being spent at stage 5. This discrepancy highlights the importance of the layout configuration in managing the participant flow and optimizing the throughput during the final stage of the RADT process. Furthermore, the mean completion time for the entire screening process was approximately 2 min for both layouts. Stages 4 (sample mixing) and 5 (result dissemination) were the most time-intensive, with the sample mixing times ranging from 29 to 31 s and the result stages varying from 46 to 62 s.

However, the average completion time for the U-shaped layout demonstrated a slight advantage over the straight-line configuration, with an average completion time of 114 s compared to 122 s, respectively. Furthermore, for the U-shaped layout and the straight-line layout, the confidence intervals for the mean time spent at each stage are illustrated in Table 1. While the differences in the time taken at each stage of the testing process between floor plans 1 and 2 were not substantial, the U-shaped layout demonstrated a slight edge in its overall efficiency.

**Figure 3:**
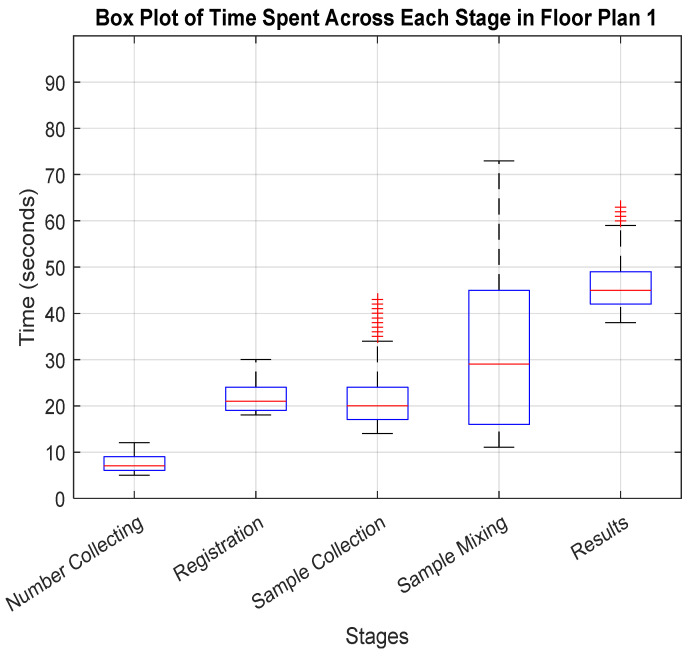
Box plot for time spent across the stages in floor plan 1 (U-shaped). Blue boxes indicate the interquartile range with the median shown by a red line. Whiskers extend to values within 1.5 times the IQR from the quartiles, showing typical data points. Points beyond this range are marked in red as outliers.

**Figure 4:**
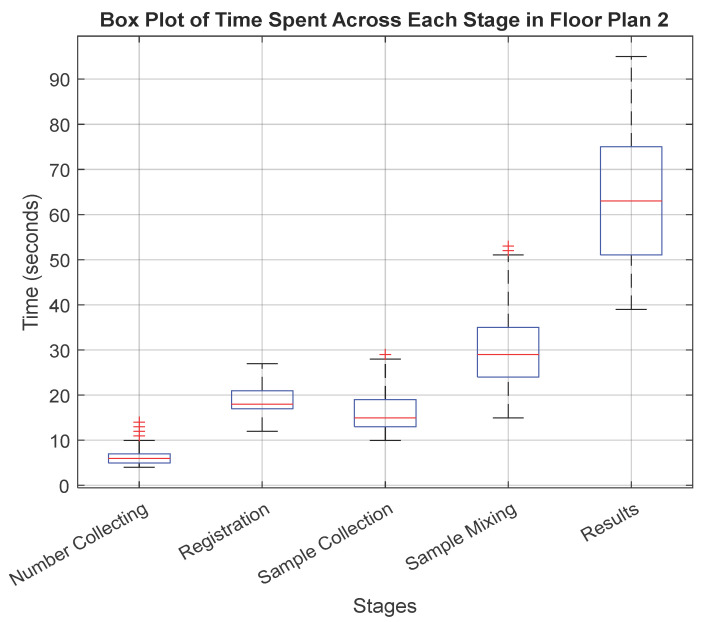
Box plot for time spent across the stages in floor plan 2 (straight line). Blue boxes indicate the interquartile range with the median shown by a red line. Whiskers extend to values within 1.5 times the IQR from the quartiles, showing typical data points. Points beyond this range are marked in red as outliers.

The detailed box plot visualizations in Figure 3 and Figure 4 provide insights into the time dynamics at each stage.

For floor plan 1, as depicted in Figure 3, the initial number collection stage is characterized by swiftness, with a median time of around 7 s and a tight interquartile range (IQR) from approximately 5 to 12 s, indicating consistent and rapid processing. The registration stage follows, with a median of about 21 s and an IQR from 18 to 30 s, displaying stability without significant outliers. In the sample collection stage, although the median time remains efficient at around 20 s, the IQR widens substantially from 14 to 43 s, suggesting variable delays, likely due to the differing sample complexities. The sample mixing stage then shows further variability, with a median time of 29 s and an even broader IQR from 11 to 73 s, highlighting pronounced inconsistencies and procedural challenges. Finally, the result stage exhibits the most significant fluctuation, with a median time of 45 s and an IQR from 38 to 63 s, plus outliers indicating sporadic delays, which could impact the overall throughput.

Switching to floor plan 2 in Figure 4, the number collection stage slightly improves, with a median time of 6 s and an IQR from 4 to 14 s, although the outliers above the upper whisker hint at occasional procedural irregularities. The registration stage shows a slight decrease in the median time to 18 s, with a narrower IQR from 12 to 27 s, suggesting a more streamlined process without outliers. However, in the sample collection stage, while the median time drops to about 15 s, the presence of outliers above the upper whisker within an IQR of 10 to 29 s indicates occasional delays. The sample mixing stage continues to be a bottleneck, with a median time near 29 s and an IQR stretching from 15 to 53 s, underscored by several outliers, pointing to significant procedural variability. Lastly, although slower, with a median time of 63 s, the result stage shows a moderate IQR from 39 to 95 s but no outliers, reflecting inherent variability without extreme deviations.

From Table 2 and Table 3, the data reveal key insights into the performance of the U-shaped and straight-line layouts across different simultaneous arrival probabilities. In the U-shaped layout, the system begins to stabilize with 12 lanes, even at higher arrival probabilities of 50% to 70%, demonstrating effective flow management. However, with only 10 lanes, the system becomes unstable as the probability of simultaneous arrivals increases beyond 20%, indicating that fewer lanes are insufficient to handle larger crowds efficiently. In contrast, the straight-line layout requires a minimum of 18 lanes to maintain stability, even under lower arrival probabilities. At 10 lanes, the system becomes unstable at just 20% simultaneous arrivals, showing that the straight-line layout demands more resources to manage congestion effectively. Even with 20 lanes, the utilization in the straight-line layout reaches 0.89 at 40% probability, suggesting more stress on the system compared to that on the U-shaped layout.

Across all of the tested scenarios, the metrics for mean waiting time, queue length, and blocking probability remained approximately zero, indicating a smooth participant flow with minimal delays between stages, likely due to the controlled nature of the experiment. The selection of simultaneous arrival probabilities reflects realistic scenarios, 50% to 70% for concerts or mass events, 20% to 40% for airport arrivals, and 10% to 30% for airport departures, ensuring that the layouts are tested under conditions that mimic real-world settings.

As the arrival rate (*λ*) is defined as the number of visitors arriving per unit of time, in our study, the average arrival rate was around 10 visitors per minute. The service rate (*μ*), representing the capacity of each testing lane to process visitors, is derived from the average screening duration. Since the average test duration across both layouts was around two minutes per visitor, this corresponds to a service rate of 0.5 visitors per minute per lane.

The traffic intensity (ρ), which quantifies the workload of the system, is calculated using the following formula [22,23,24]:

(1) $$\rho =\frac{\lambda }{\left(c\times \mu \right)}.$$

where *c* is the number of lanes (or servers) available. This formula helps evaluate the utilization of the system, showing the fraction of time for which the servers are expected to be busy.

According to queuing theory, the following conditions hold for traffic intensity (ρ) [22]:

ρ < 1: The system has adequate capacity to handle the incoming rate without causing excessive queuing.

ρ = 1: The system is at full capacity, creating a risk of queue buildup if the arrival rate increases or the service rate decreases.

ρ > 1: The system is overloaded, causing queues to grow indefinitely.

These conditions highlight the importance of keeping ρ < 1 to prevent unbounded queues and ensure finite waiting times. In this study, as we employed the *MAP/PH/c* model to capture more realistic arrival patterns and service time distributions, the model provided a more accurate representation of the testing process than simpler queuing models.

Finally, the Pollaczek–Khinchine formula [22,23,24] helps us calculate the average waiting time in the queue (*Wq*) for a system with ρ < 1:

(2) $$Wq=\frac{Pb}{\lambda \cdot (1-\rho )}.$$

where *Pb* is the blocking probability, calculated using the Erlang-B formula. This formula enables us to predict the waiting times under different system loads and plan for optimal operations.

Additionally, throughput, or the rate at which participants are served, is calculated using the following formula from the *MAP/PH/c* model [22,23,24]:

(3) Throughput=λ⋅(1−Pb).

This accounts for any blocked arrivals and provides an accurate estimate of the system’s capacity to serve participants efficiently.

The *MAP/PH/c* model was implemented in our analysis using MATLAB as follows:

 *function [Wq, Lq, rho, Pb, throughput] = map_ph_c_analysis(D0, D1, c)*

   *% MAP/PH/c Model Analysis*

   *% D0: Infinitesimal generator matrix (no arrival transitions)*

   *% D1: Infinitesimal generator matrix (arrival transitions)*

   *% c: Number of servers (e.g., testing lanes)*

   *lambda = sum(D1(:)); % Sum of all elements in D1 (arrival rates)*

   *mu =* *−* *1/sum(diag(D0)); % Service rate from D0*

   rho = lambda/(c * mu); % Traffic intensity

   *if rho >= 1*

    *error(’System is unstable: rho >= 1. Increase the number of servers.’);*

   *end*

   *Pb = (rho^c/factorial(c))/sum((rho.^(0:c))./factorial(0:c)); % Blocking probability*

   *Wq = (Pb/(1 - rho)) / lambda; % Average waiting time in queue*

   Lq = lambda * Wq; % Average queue length

   throughput = lambda * (1 - Pb); % Throughput

   *fprintf(’Server Utilization (rho): %.2f\n’, rho);*

   *fprintf(’Mean Waiting Time (Wq): %.2f seconds\n’, Wq);*

   *fprintf(’Average Queue Length (Lq): %.2f\n’, Lq);*

   *fprintf(’Blocking Probability (Pb): %.4f\n’, Pb);*

   *fprintf(’Throughput: %.2f participants per second\n’, throughput);*

 *end*

This model refines its predictions by accounting for variable arrival patterns and service times in both layouts.

## 4. Discussion

The *t*-test results indicated no significant differences in the processing times between stages 1 through 4 across the two floor plans (*p* > 0.05), suggesting that these stages were unaffected by the spatial configurations. However, a significant difference was observed at stage 5 (*p* < 0.05), indicating that the layout configuration has a notable impact on the efficiency of the dissemination of results. The U-shaped layout likely provided a smoother participant flow and better resource management during this stage, minimizing delays. In contrast, the straight-line layout may have contributed to bottlenecks, resulting in slightly longer processing times at stage 5. These findings suggest that while there is a similar performance at earlier stages, the overall layout plays a critical role in the final stage, influencing the cumulative testing experience by managing congestion and throughput effectively.

Both floor plans achieved an approximate mean completion time of 2 min for the entire screening process. Notably, stages 4 (sample mixing) and 5 (result dissemination) consistently required more time across both setups, with the variations observed likely influenced by the manual handling and processing inherent to these stages. Despite the similar median times, the U-shaped layout demonstrated a marginally faster average completion time (114 s) compared to the straight-line setup (122 s). This slight advantage is attributed to the U-shaped design facilitating better movement and less crowding, enhancing the overall flow efficiency.

The 95% confidence intervals for the mean times at each stage were generally narrower in the U-shaped layout, indicating less variability and greater consistency in the processing times compared to the straight-line configuration.

The arrival rate (*λ*) averaged about ten visitors per minute. In contrast, the service rate (*μ*), determined based on the average duration it took for each visitor to complete the screening process, approximately two minutes per visitor, was 0.5 visitors per minute per lane. 

Regarding the number of lanes, we assessed the system’s capacity to manage incoming visitor rates without excessive queuing. Using the *MAP/PH/c* model, which captures realistic arrival patterns and phase-type service times better, we aimed to maintain a traffic intensity (*ρ*) of less than 1 to ensure system stability and avoid bottlenecks. The equation $c=\frac{\lambda }{u}$ was used as a baseline, but additional adjustments were made based on the *MAP/PH/c* model’s results to ensure the optimal flow. Based on these calculations, the model suggested that approximately 18 to 20 lanes are necessary to handle the arrival rate efficiently under different simultaneous arrival probabilities. This ensures that the system can operate smoothly without excessive wait times or queues building up.

The U-shaped layout appears more robust, requiring fewer lanes (12) to handle higher simultaneous arrival probabilities compared to the straight-line layout (which needs at least 18 lanes for stability). This suggests that the layout design significantly impacts throughput and utilization, and the choice of layout must consider the nature of the event and the expected arrival pattern to ensure smooth operation.

We acknowledge several limitations in this study that can be addressed in future research:

1. Sample Size and Generalizability:
  While this study involved 500 participants, a larger sample size across multiple events would provide more robust insights and increase the generalizability of the results. Future studies should include more participants and varied event types to validate the findings further.
2. Single Test Type:
  This study focused solely on Abbott Panbio COVID-19 rapid antigen tests. Expanding future research to include different types of diagnostic tests would offer a broader understanding of how different tests perform under similar conditions.
3. A Controlled Environment vs. Real-World Settings:
  This study was conducted in a controlled test center environment, which may not fully capture the complexities of real-world mass events. Future studies will aim to validate these findings in real-world event settings to assess any operational challenges that may arise.
4. Limited Exploration of Variability:
  While we evaluated the performance under different simultaneous arrival probabilities, real events may introduce further variability into the arrival patterns and participant behavior. Future research will simulate more complex scenarios to reflect actual event conditions better.
5. The Static Queueing Model:
  Although we used the *MAP/PH/c* model to improve upon the simpler *M/M/c* queueing model, further refinement is possible. Incorporating real-time data and dynamic adjustments to the number of lanes based on fluctuating arrival rates could enhance future models’ accuracy.
6. Focus on Two Layouts Only:
  We only analyzed two floor layouts (U-shaped and straight-line). Future studies can explore additional configurations and their impact on throughput and efficiency.

In future work, we will address these limitations by employing larger sample sizes, exploring additional test types, conducting studies in real-world event settings, and refining our modeling approach with more dynamic elements. This will allow us to provide deeper insights into optimizing rapid testing operations at mass events.

## 5. Conclusions

This study demonstrates the potential of employing rapid antigen tests (RADTs) effectively in large-scale public events, emphasizing the critical role of the spatial layouts and operational strategies in optimizing the testing throughput. Our findings reveal that a U-shaped layout offers a better performance under higher simultaneous arrival probabilities, stabilizing with fewer lanes compared to the straight-line configuration. Specifically, the U-shaped layout achieved stability with 12 lanes, whereas the straight-line layout required at least 18 lanes to handle the same arrival rates efficiently.

The results also indicate that the mean waiting time, queue length, and blocking probability were minimal across all configurations, underscoring the practicality of implementing these testing strategies in mass gatherings. By employing the *MAP/PH/c* queuing model, we identified the optimal number of lanes to maintain system efficiency and avoid bottlenecks. These insights offer valuable guidance for event organizers and health authorities in planning resource allocation and designing layouts for mass testing events.

While this study validated the feasibility of using RADTs in controlled environments, future research should explore real-world events with larger sample sizes and diverse test types to refine the findings further. This study has contributed to the development of effective testing protocols, ensuring rapid and streamlined screening processes essential for public health management at mass gatherings.

## Figures and Tables

**Table 1:** U-shaped and straight-line floor plan stage statistics.

Stage	Process	U-Shaped			Straight-Line		
		Mean Time (s)	Standard Deviation (s)	95% CI (s)	Mean Time (s)	Standard Deviation (s)	95% CI (s)
Stage 1	Queue Number Collection	7.8	2.0	7.62 to 7.98	6.8	2.2	6.61 to 6.99
Stage 2	Registration	21.9	3.3	21.61 to 22.19	18.6	3.1	18.33 to 18.87
Stage 3	Sample Collection	21.1	5.4	20.63 to 21.57	16.5	4.6	16.10 to 16.90
Stage 4	Sample Mixing	31.4	15.7	30.02 to 32.78	29.8	8.4	29.06 to 30.54
Stage 5	Result	46.1	5.1	45.65 to 46.55	62.6	14.8	61.30 to 63.90
**Average Total Time**		114.2 s			122.7 s		
**Total Time Standard Deviation**		17.8 s			17.6 s		

**Table 2:** Utilization and throughput for U-shaped layout at different arrival probabilities.

U-Shaped							
Simultaneous Arrival Probabilities		10%	20%	30%	40%	50%	70%
**10 lanes**	Utilization	0.98	system unstable				
	Throughput	0.09					
**12 lanes**	Utilization	0.81	0.96	0.74	0.95	0.96	0.77
	Throughput	0.11	0.10	0.09	0.09	0.09	0.08
**20 lanes**	Utilization	0.49	0.57	0.44	0.57	0.58	0.46
	Throughput	0.18	0.17	0.17	0.16	0.15	0.14

**Table 3:** Utilization and throughput for straight-line layout at different arrival probabilities.

Straight Line							
Simultaneous Arrival Probabilities		10%	20%	30%	40%	50%	70%
**10 lanes**	Utilization	0.95	system unstable				
	Throughput	0.08					
**18 lanes**	Utilization	0.41	0.57	0.47	0.99	0.41	0.59
	Throughput	0.15	0.15	0.14	0.13	0.13	0.11
**20 lanes**	Utilization	0.47	0.51	0.42	0.89	0.37	0.53
	Throughput	0.16	0.16	0.15	0.14	0.13	0.12

## Data Availability

The data presented in this study are available on request from the corresponding author. The data are not publicly available for privacy reasons.

## References

[1] Peeling R.W., Heymann D.L., Teo Y.-Y., Garcia P.J. (2022). Diagnostics for COVID-19: Moving from pandemic response to control. Lancet.

[2] McAloon C.G., Dahly D., Walsh C., Wall P., Smyth B., More S.J., Teljeur C. (2022). Potential Application of SARS-CoV-2 Rapid Antigen Diagnostic Tests for the Detection of Infectious Individuals Attending Mass Gatherings—A Simulation Study. Front. Epidemiol..

[3] Peeling R.W., Olliaro P.L., Boeras D.I., Fongwen N. (2021). Scaling up COVID-19 rapid antigen tests: Promises and challenges. Lancet Infect. Dis..

[4] Wertenauer C., Michael G.B., Dressel A., Pfeifer C., Hauser U., Wieland E., Mayer C., Mutschmann C., Roskos M., Wertenauer H.-J. (2022). Diagnostic Performance of Rapid Antigen Testing for SARS-CoV-2: The COVID-19 AntiGen (COVAG) study. Front. Med..

[5] Khan A., Alsofayan Y., Alahmari A., Alowais J., Algwizani A., Alserehi H., Assiri A., Jokhdar H. (2021). COVID-19 in Saudi Arabia: The national health response. East. Mediterr. Health J..

[6] Pondaven-Letourmy S., Alvin F., Boumghit Y., Simon F. (2020). How to perform a nasopharyngeal swab in adults and children in the COVID-19 era. Eur. Ann. Otorhinolaryngol. Head Neck Dis..

[7] Filchakova O., Dossym D., Ilyas A., Kuanysheva T., Abdizhamil A., Bukasov R. (2022). Review of COVID-19 testing and diagnostic methods. Talanta.

[8] Xie J.-W., He Y., Zheng Y.-W., Wang M., Lin Y., Lin L.-R. (2022). Diagnostic accuracy of rapid antigen test for SARS-CoV-2: A systematic review and meta-analysis of 166,943 suspected COVID-19 patients. Microbiol. Res..

[9] Skittrall J.P., Wilson M., Smielewska A.A., Parmar S., Fortune M.D., Sparkes D., Curran M.D., Zhang H., Jalal H. (2021). Specificity and positive predictive value of SARS-CoV-2 nucleic acid amplification testing in a low-prevalence setting. Clin. Microbiol. Infect..

[10] Saidani M., Kim H., Kim J. (2021). Designing optimal COVID-19 testing stations locally: A discrete event simulation model applied on a university campus. PLoS ONE.

[11] Kumar R. (2020). Modeling and Simulation Concepts.

[12] Green L.V. (2006). Patient Flow: Reducing Delay in Healthcare Delivery.

[13] Smith J.S., Sturrock D.T. (2021). Simio and Simulation: Modeling, Analysis, Applications, Sewickley.

[14] Little J.D., Graves S.C. (2008). Building Intuition: Insights from Basic Operations Management Models and Principles.

[15] Shortle J.F., Thompson J.M., Gross D., Harris C.M. (2018). Fundamentals of Queueing Theory.

[16] Turki A., Raml E. (2023). Enhancing Pediatric Adnexal Torsion Diagnosis: Prediction Method Utilizing Machine Learning Techniques. Children.

[17] (2021). Abbott COVID-19 Ag Rapid Test Device. https://dam.abbott.com/en-gb/panbio/120007883-v1-Panbio-COVID-19-Ag-Nasal-AsymptomaticSe.pdf.

[18] Autodesk Revit: BIM Software to Design and Make Anything. Autodesk. https://www.autodesk.com/products/revit/overview?term=1-YEAR&tab=subscription.

[19] Google LLC Google Sheet. https://docs.google.com/spreadsheets/u/0/?usp=sheets_ald.

[20] R Core Team (2024). R: A Language and Environment for Statistical Computing.

[21] The MathWorks, Inc. (2024). *MATLAB R2022b*; Natick, MA, USA. https://www.mathworks.com.

[22] Gerontidis I.I., Kalashnikov V.V. (1995). Mathematical Methods in Queuing Theory.

[23] Haghighi A.M., Mishev D. (2013). Difference and Differential Equations with Applications in Queueing Theory.

[24] Wiler J.L., Bolandifar E., Griffey R.T., Poirier R.F., Olsen T. (2013). An emergency department patient flow model based on queueing theory principles. Acad. Emerg. Med..

